# No Association Between G1246A Polymorphism in *HCRTR2* Gene and Risk of Cluster Headache: Evidence From an Updated Meta-Analysis of Observational Studies

**DOI:** 10.3389/fgene.2020.560517

**Published:** 2020-12-03

**Authors:** Jiao Yang, Si-yi Yu, Jie Yang, Jing Kong, Fan-rong Liang, Zheng-tao Lv

**Affiliations:** ^1^The 3rd Teaching Hospital, Chengdu University of Traditional Chinese Medicine, Chengdu, China; ^2^Department of Orthopedics, Tongji Medical College, Tongji Hospital, Huazhong University of Science and Technology, Wuhan, China

**Keywords:** cluster headache (CH), *HCRTR2*, meta-analysis, G1246A polymorphism, rs2653349

## Abstract

**Background:** The hypocretin receptor 2 (*HCRTR2*) gene may play a pathological role in cluster headache (CH). However, the conclusions of published reports on the relationship between the G1246A polymorphism (rs2653349) in the *HCRTR2* gene and risk of CH remain controversial. This purpose of this article is to comprehensively study the current evidence and assess the association between G1246A polymorphism (rs2653349) in the *HCRTR2* gene and risk of CH.

**Materials and Methods:** Four electronic databases—ISI Web of Science, CNKI, PubMed, and EMBASE—were comprehensively searched on August 2020 to find and pinpoint all observational articles related to this study. The association between G1246A polymorphism in the *HCRTR2* gene and risk of CH under five different genetic models was evaluated based on the summary odds ratio and corresponding 95 confidence interval (95% CI). Methodological quality was assessed based on the Newcastle–Ottawa Scale (NOS). To assist the analysis, RevMan 5.3 software was used to perform subgroup and sensitivity analyses. Egger's and Begg's tests were then conducted to evaluate and assess publication bias. Finally, a meta-regression was carried out by residual (restricted) maximum likelihood (REML).

**Results:** Eight observation studies containing 3,161 healthy controls and 1,964 patients with CH were identified and to be used for the meta-analysis. With methodological quality NOS assessment, the incorporated studies showed an average score of 6.4 stars. The pooled data didn't support the association between G1246A polymorphism in the *HCRTR2* gene and CH vulnerability in the overall population (OR: 0.85, 95% CI 0.69, 1.03; *p* = 0.10). Subgroup analysis by ethnicity showed no significant association between G1246A and CH in either Caucasians (OR: 0.89, 95% CI 0.77, 1.01; *p* = 0.08) or Asians (OR: 1.65, 95% CI 0.80, 3.41; *p* = 0.18). The robustness of the conclusion was tested and confirmed with the leave-one-out sensitivity analysis. Meta-regression analysis showed that chronological order of publication appeared to be significantly associated with the heterogeneity (*t* = 2.47, *p* = 0.039; residual *I*^2^ = 0%, adjusted *R*^2^ = 100%).

**Conclusion:** Our present study showed that the G1246A polymorphism in the *HCRTR2* gene did not appear to be an accomplice and associated with CH predisposition among either the Asian or Caucasian population.

## Introduction

Cluster headache (CH) is a severe neurovascular disease characterized by recurring short-lasting attacks of excruciating unilateral pain (Leone and Proietti Cecchini, 2017). Based on the International Classification of Headache Disorders, 3rd edition (ICHD-III), CH is one of the trigeminal autonomic cephalalgias (TACs) accompanied by cranial autonomic symptoms, such as nasal congestion, runny nose (rhinorrhea), tears, and eye congestion [Headache Classification Committee of the International Headache Society (IHS), 2018]. Epidemiological studies have shown that a lifetime prevalence of CH was 0.12% for all adults of both genders, and the 1-year prevalence of CH was 0.05% (Fischera et al., 2008). Most patients with CH experienced substantial burdens at work (Choi et al., 2018) and the total direct cost for CH is greater than $2.8 billion/year (Choong et al., 2018). CH inevitably imposes a heavy burden to both society and individuals.

**Figure 1:**
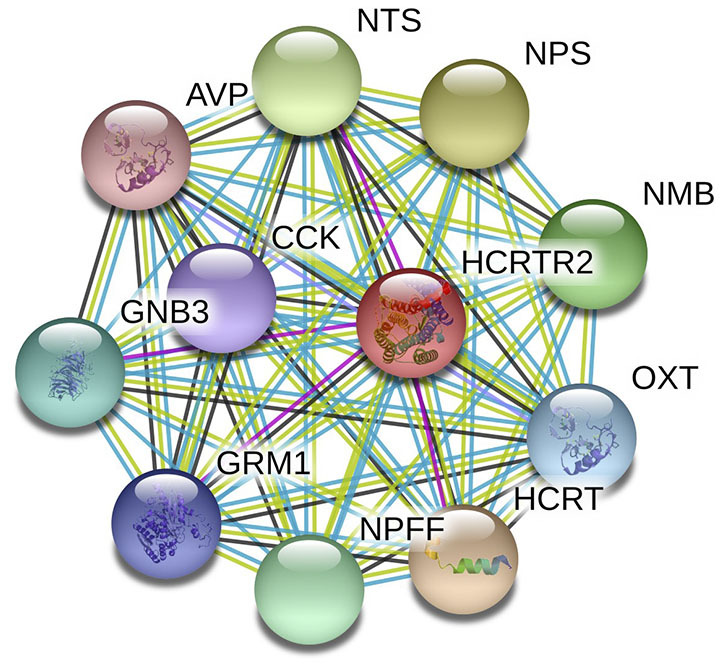
PPI network of HCRTC2 and its closest functional partners.

The underlying etiopathogenesis of CH remains largely indeterminate. It is reported that CH is driven by various factors, including race (Rozen et al., 2001; Mengistu and Alemayehu, 2013), gender (Rozen et al., 2001; Chung et al., 2019), and age (Manzoni et al., 2016, 2019) as well as genetics (Russell, 2004; Cruz et al., 2013). Among all the factors, the genetic factors showed strong association with the occurrence of CH Studies have discovered a familial aggregation of CH (Cruz et al., 2013). Compared to the general population, first-degree relatives have a 5–18 times higher risk, and in second-degree relatives, 1–3 times increased risk have been observed. Many genes with a huge number of gene polymorphisms have been classified to have covert risk alleles for CH susceptibility and vulnerability. Presently, G protein beta 3 subunit (*GNB3*) (Papasavva et al., 2020), alcohol dehydrogenase 4 (*ADH4*) (Fourier et al., 2016), and various candidate genes like hypocretin receptor type 2 (*HCRTR2*) (Fourier et al., 2019; Papasavva et al., 2020) have been widely reported to be related to CH. Figure 1 shows the *HCRTR2* protein–protein interaction (PPI) network and its closest functional protein partners. From the figure, a few proteins that interact and collaborate with *HCRTR2* are also associated with the pathogenesis of CH, including GNB3 (Papasavva et al., 2020), neuropeptide FF-amide peptide (NPFF) (Zhao et al., 2016), and hypocretin (HCRT) (Barloese et al., 2014). The *HCRTR2* gene, located on chromosome 6p12.1, consists of seven exons and encodes for a G-protein coupled receptor that is exclusively expressed in the brain (Sakurai et al., 1998). The involvement of hypocretins in the transmission of pain and in autonomic and neuroendocrine functions is associated with the pathogenesis of CH (Mobarakeh et al., 2005). Therefore, the G1246A polymorphism in *HCRTR2* could be a plausible candidate locus that contributes to the pathogenesis of CH.

A large study conducted in Germany showed that homozygous carriers of the G allele had a twofold increased risk for CH compared to heterozygous or homozygous carriers of the A allele (Schürks et al., 2006). However, subsequent replication studies that intended to validate and endorse the association brought in different and conflicting results (Weller et al., 2015; Fan et al., 2018; Fourier et al., 2019). This might be due to the limited sample size, racial differences, inadequate statistical power, or inconsistent definitions of CH, along with other unknown or undetected variations. Previous meta-analyses have been carried out, and reported the positive association between rs2653349 and risk of CH (Rainero et al., 2007; Weller et al., 2015). Considering the emergence and development of novel evidence on the association of G1246A polymorphism of the *HCRTR2* gene and risk of CH, we performed the present systematic review and meta-analysis. This gave a more conclusive and accurate association between G1246A polymorphism of the *HCRTR2* gene and CH predisposition.

## Methods

This review was conducted in accordance with the Meta-Analysis of Observational Studies in Epidemiology (MOOSE) guidelines (Stroup et al., 2000).

### Literature Search Strategy

Four electronic databases—ISI Web of Science, CNKI, PubMed, and EMBASE—were comprehensively searched from their initiation up to December 2019 without any language restriction. Before submission of this paper, these databases were searched again so that no newly published articles were missed (the last literature search was performed in August 2020). A combination of Medical Subject Headings (MeSH) alongside free terms was utilized to increase the sensitivity of the literature search. For English databases, we adopted the following search strings: [“Cluster Headache” (Mesh) or cluster headache or ciliary neuralgia or neuralgic migraine or histamine cephalgias or Horton Syndrome or trigeminal autonomic cephalalgia] and [“Polymorphism, Single Nucleotide” (Mesh) or Single Nucleotide Polymorphism or polymorphism or SNP or SNPs] and (*HCRTR2* or hypocretin receptor 2 or orexin 2 receptor or OX2R). Last, the keyword strings (HCRTR2) and (Duo Tai Xing) and (Tou Tong) were used for the CNKI electronic database. The reference list of related studies was also manually searched for additional eligible studies.

### Eligibility Criteria

Studies that met the following inclusion criteria were included: (1) subjects in CH groups should be patients diagnosed with CH according to well-established guidelines, such as the ICHD-II criteria; (2) control subjects should be defined as healthy subjects without a history of CH; (3) observational studies (case control study or cohort study) on humans; (4) the polymorphism of interest was rs2653349 in the *HCRTR2* gene; (5) the primary outcome was the relationship between *HCRTR2* polymorphism and risk of CH, which must be shown by odds ratio (OR) and the associated 95% confidence interval (95% CI), which comes from the original study or built on the allele frequencies of the variant calculations. Editorials, case reports, case series, *in vitro* experiments, and animal studies were eliminated and removed. If there were numerous studies that gave overlying outcomes and data, the most comprehensive one would be used and included in the meta-analysis. If there was only an abstract without a full paper, we would make three attempts to reach the authors by e-mail for their raw data. In the event that the authors did not respond to our e-mail, these abstracts would be regretfully dropped and discarded.

### Quality Assessment

The methodological quality of eligible studies was evaluated according to the Newcastle–Ottawa Scale (NOS) for observational studies (Peterson et al., 2011). An assessment system, “star system,” was used to assess each study based on three aspects. The three aspects were the study group selections, the comparability of these groups, and ascertainment of the outcome of interest. Each study was then scored on a scale from 0 to 9 on their methodological quality. The scores were then divided into low (0–3 points), moderate (4–6 points), and high (7–9 points). Two reviewers (JY and SY) independently evaluated each included study, then the results were compared. Should there be any disagreement between the two investigators, the disagreement would be discussed to achieve a mutual consensus. In the rare event that mutual consensus could not be reached, a third reviewer (ZL) would join the discussion to reach mutual consensus.

### Data Extraction

Two reviewers (JY and SY) independently performed data extraction of all included studies according to the predetermined eligibility criteria. The data extraction would be done with the standard data collection form: first author's name, publication year, origin country, subjects' ethnicity, source of the subjects, size of the sample, diagnostic criteria of CH, genotype distribution in CH and control groups, Hardy–Weinberg equilibrium (HWE), calculated OR, and 95% CI of individual study.

### Quantitative Synthesis

HWE for the control participants was assessed using the chi-square test to review its goodness of fit. The estimated genetic effect was presented as OR and corresponding 95% CI based on the genotype count extracted from the included studies to evaluate and assess the correlation between rs2653349 and risk of CH. In this review, a meta-analysis under five different genetic models was conducted: dominant model (AA + AG vs. GG), recessive model (AA vs. AG + GG), allelic model (A vs. G), homozygote model (AA vs. GG), and heterozygote model (AG vs. GG). The significance threshold for the meta-analyses was estimated by the Bonferroni correction (0.05/5 = 0.01) (Armstrong, 2014). The *Q*-test and the Higgins *I*^2^ test were used to estimate the intrastudy heterogeneity. *p* > 0.1 and *I*^2^ <50% indicated acceptable variability among the included studies (Higgins et al., 2003). However, regardless of the magnitude of heterogeneity across studies, we used the random effect model for quantitative synthesis due to the presence of anticipated heterogeneity across studies (Mantel and Haenszel, 1959; DerSimonian and Laird, 1986). We hypothesized that the true genetic effect of HCRTR2 polymorphism varied among different populations.

An ethnicity subgroup analysis was performed to check the impact of rs2653349 in subjects of different ethnicities. In order to examine the robustness of the summary risk estimate, the leave-one-out sensitivity analysis was used. This analysis tested the pooled results by reassessing the result effect by removing each study one by one. The RevMan 5.3 software provided the forest plots (Copenhagen: The Nordic Cochrane Centre, The Cochrane Collaboration, 2014). Egger's regression test and Begg's rank correlation test were used to estimate the publication bias using Stata version 12.0 (StataCorp LP, College Station, Texas). The outcome of *p* < 0.05 illustrates significant publication bias (Egger et al., 1997). In case of significant heterogeneity across studies, a meta-regression by ethnicity (Asian and Caucasian), sample size (*n* < 500 and *n* ≥ 500), methodological quality (low, moderate, or high quality) and diagnostic criteria (ICHD-II or ICHD-III) was performed to identify the potential source of heterogeneity. Additionally, meta-regression was performed to test whether the discrepancy between results of the first several studies (published before 2010) and subsequent studies (published after 2010) appeared genuine (Salanti et al., 2005).

## Results

### Literature Search

**Figure 2:**
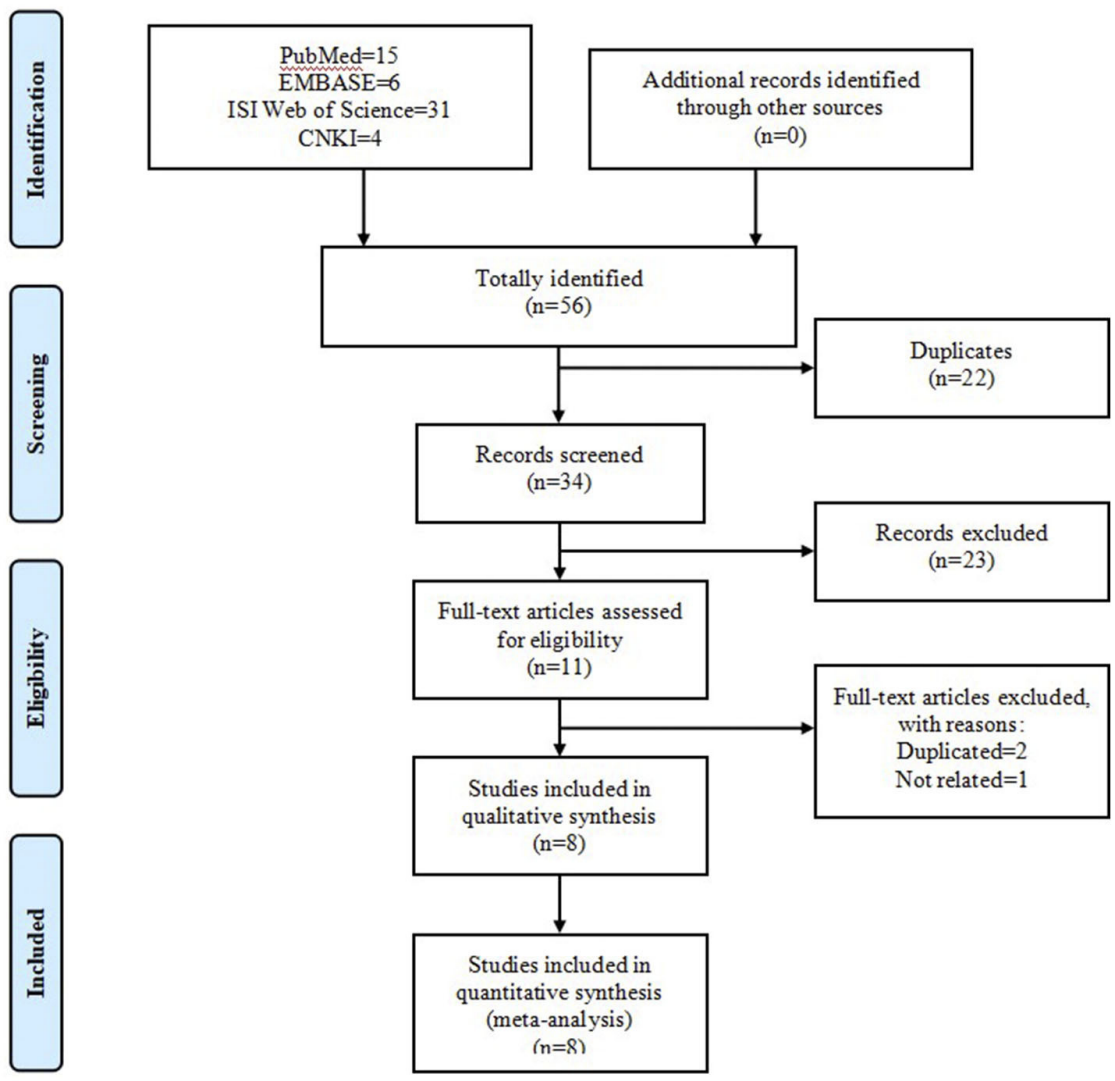
Flow chart of literature search and screen.

The initial search of four online databases yielded 66 records, comprising 15 from PubMed, 8 from EMBASE, 36 from ISI Web of Science, and 7 from CNKI. No additional record was identified through other sources. After the first stage of scanning, 31 duplicated records were excluded. Of the remaining 35 records, a further 24 citations were eliminated after title and abstract screening. Among the remaining 11 articles, 1 was considered unrelated based on predefined inclusion and exclusion criteria and was removed, and 2 were excluded because of duplicated data. Ultimately, eight articles (Baumber et al., 2006; Schürks et al., 2006; Rainero et al., 2008; Weller et al., 2015; Zarrilli et al., 2015; Fan et al., 2018; Fourier et al., 2019; Papasavva et al., 2020) were included in the systematic review and meta-analysis. Figure 2 shows the process of literature selection.

### Main Characteristics and Methodological Quality

Table 1 shows the main characteristics of the studies that were incorporated. Eight studies, with a total of 1,964 CH patients and 3,161 healthy controls, were included in this meta-analysis. All the articles were published in English between 2006 and 2020. One of the studies was carried out in China (Fan et al., 2018); two in Italy (Rainero et al., 2008; Zarrilli et al., 2015); one in the UK, Denmark, and Sweden (Baumber et al., 2006); one in Sweden (Fourier et al., 2019); one in Greece (Papasavva et al., 2020); one in Germany (Schürks et al., 2006); and one in the Netherlands (Weller et al., 2015). All the studies were conducted in Caucasian populations, expect one in Asian populations (Fan et al., 2018). One of the studies (Baumber et al., 2006) was a case-control cohort study in design and the rest were case-control studies in design. The sample size of the individual studies ranged from 54 to 575 for cases, and 72 to 874 for controls. All of the subjects were collected from hospitals, except in one case from websites (Weller et al., 2015). All CH patients were diagnosed according to well-established diagnostic criteria including the International Classification of Headache Disorders, 3rd edition (ICHD-III) [Headache Classification Committee of the International Headache Society (IHS) 2018]; International Classification of Headache Disorders, 2nd edition (ICHD-II) [Headache Classification Committee of the International Headache Society (IHS) 2004]; and International Classification of Headache Disorders (ICHD-III beta) [Headache Classification Committee of the International Headache Society (IHS) 2013]. None of the included studies deviated from the HWE with the exception of the study by Zarrilli et al. [2015]. The genotype distribution of the case and control groups is summarized in Table 1. With the methodological quality NOS assessment, the incorporated studies showed an average score of 6.4 stars. The response of each individual study to NOS is shown in Table 2.

**Table 1:** Main characteristics of included studies.

**Study**	**Country**	**Ethnicity**	**Sample size (CH/control)**	**Source of subjects**	**Diagnostic criteria**	**CH**			**Control**			**HWE**
						**GG**	**GA**	**AA**	**GG**	**GA**	**AA**	
Baumber et al. (2006) (1)	UK	Caucasian	63/89	Hospital-based	ICHD-II	41	20	2	57	27	5	0.46
Baumber et al. (2006) (2)	Denmark	Caucasian	96/72	Hospital-based	ICHD-II	56	38	2	37	31	4	0.44
Baumber et al. (2006) (3)	Sweden	Caucasian	98/106	Hospital-based	ICHD-II	68	26	4	67	32	7	0.25
Fan et al. (2018)	China	Asian	112/192	Hospital-based	ICHD-III beta	98	13	1	176	16	0	0.83
Fourier et al. (2019)	Sweden	Caucasian	517/581	Hospital-based	ICHD-III	332	168	17	385	174	22	0.91
Papasavva et al. (2020)	Greece	Caucasian	114/570	Hospital-based	ICHD-III	91	21	2	451	109	10	0.53
Rainero et al. (2008)	Italy	Caucasian	109/211	Hospital-based	ICHD-II	103	4	2	163	43	5	0.58
Schürks et al. (2006)	Germany	Caucasian	226/266	Hospital-based	IHS-1998, ICHD-II	173	46	7	166	93	7	0.15
Weller et al. (2015)	The Netherlands	Caucasian	575/874	Web-based	ICHD-II	351	206	18	522	307	45	0.99
Zarrilli et al. (2015)	Italy	Caucasian	54/200	Hospital-based	ICHD-III beta	43	9	2	165	27	8	0.0002

*Study conducted by Baumber et al. (2006) consists of three parts which was carried out in UK, Denmark and Sweden, respectively. CH, cluster headache; ICHD, International Classification of Headache Disorders; HWE, Hardy-Weinberg Equilibrium*.

**Table 2:** Quality assessment of included studies based on of the Newcastle-Ottawa Scale.

**Item/study**	**Baumber et al. [2006]**	**Fan et al. [2018]**	**Fourier et al. [2019]**	**Papasavva et al. [2020]**	**Rainero et al. [2008]**	**Schürks et al. [2006]**	**Weller et al. [2015]**	**Zarrilli et al. [2015]**
Adequate definition of cases	*	*	*	*	*	*	*	*
Representativeness of cases	–	–	–	–	*	–	–	–
Selection of control subjects	–	–	–	–	–	*	–	–
Definition of control subjects	*	*	*	*	*	*	–	*
Control for important factor or additional factor	*	**	*	*	**	*	*	*
Exposure assessment	*	*	*	*	*	*	*	*
Same method of ascertainment for all subjects	*	*	*	*	*	*	*	*
Non-response rate	*	*	*	*	*	*	*	*

*A study could be awarded a maximum of one star for each item except for the item “Control for important factor or additional factor”*.

*The definition/explanation of each column of the Newcastle-Ottawa Scale is available from http://www.ohri.ca/programs/clinical_epidemiology/oxford.asp*.

### Meta-Analysis

**Figure 3:**
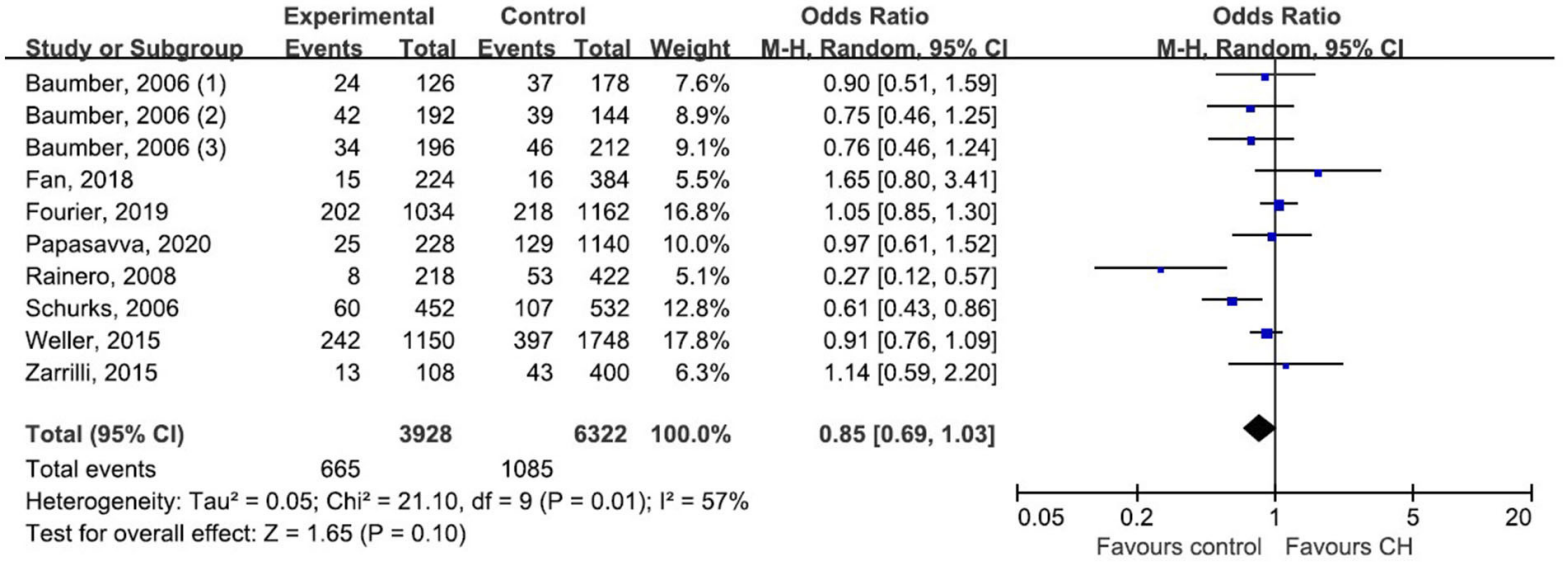
Forest plot of G1246A in the HCRTR2 gene and risk of CH using a random effect model.

We pooled data from each individual study using five genetic models: dominant model, recessive model, allelic model, homozygote model, and heterozygote model. But none of those genetic models suggested a significant association between rs2653349 and risk of CH, with ORs ranging from 0.73 to 0.85 and the 95% CIs ranging from 0.52 to 1.11. Considering the allele model is more representative than other models, we only presented the association under the allele model where the counts of allele A were compared with allele G. Due to moderate heterogeneity among the included studies (*p* = 0.01, *I*^2^ = 57%), statistical analysis was done with the random-effect model. The combined OR and corresponding 95% CI gave an outcome that rs2653349 and risk of CH in the overall population (OR 0.85, 95% CI 0.69, 1.03; *p* = 0.10) have no significant association statistically (Figure 3). Considering that multiple hypotheses testing were suspected, we performed the Bonferroni correction with the *p* threshold value of 0.01.

### Subgroup Analysis and Publication Bias

**Figure 4:**
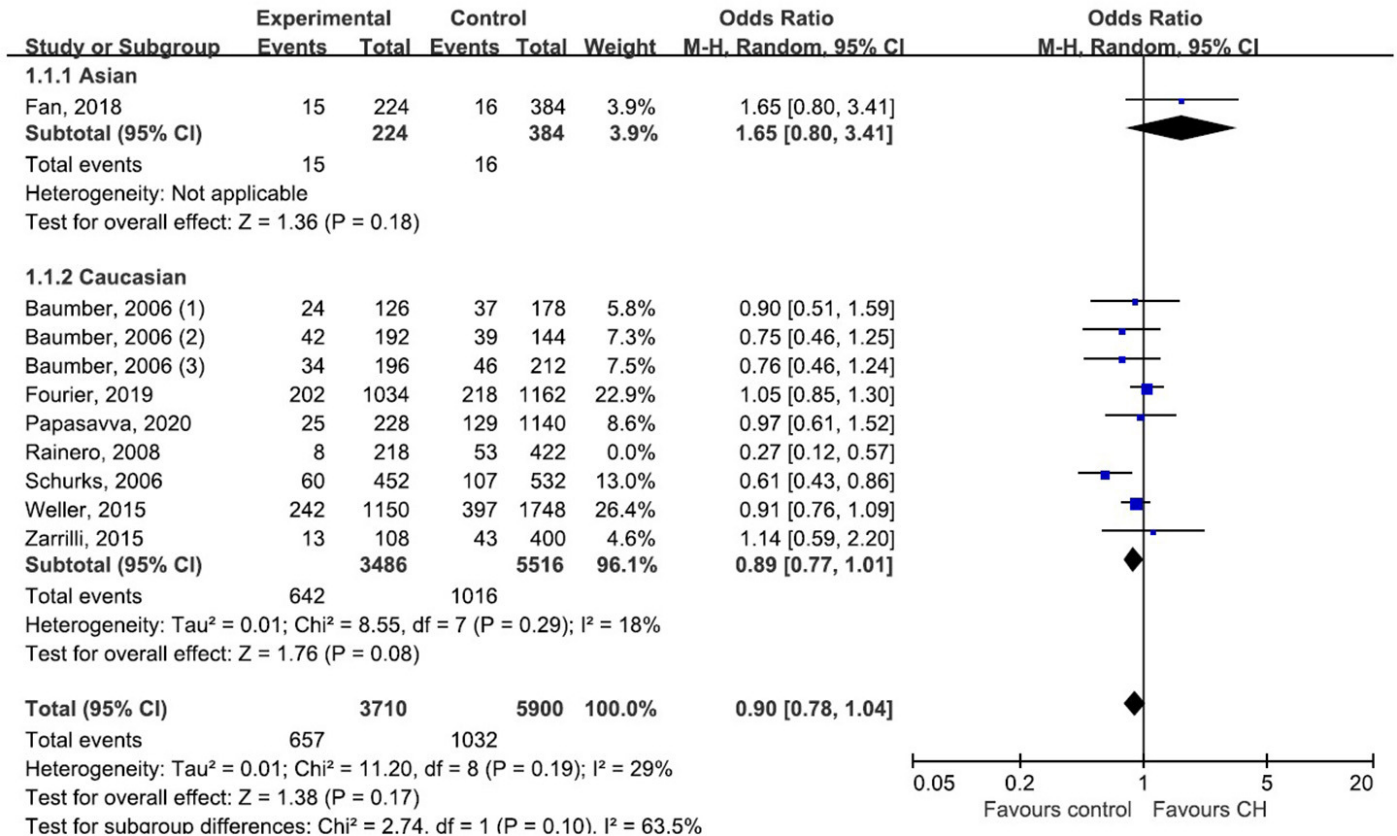
Subgroup analysis of association between G1246A polymorphism in the HCRTR2 gene and CH.

**Figure 5:**
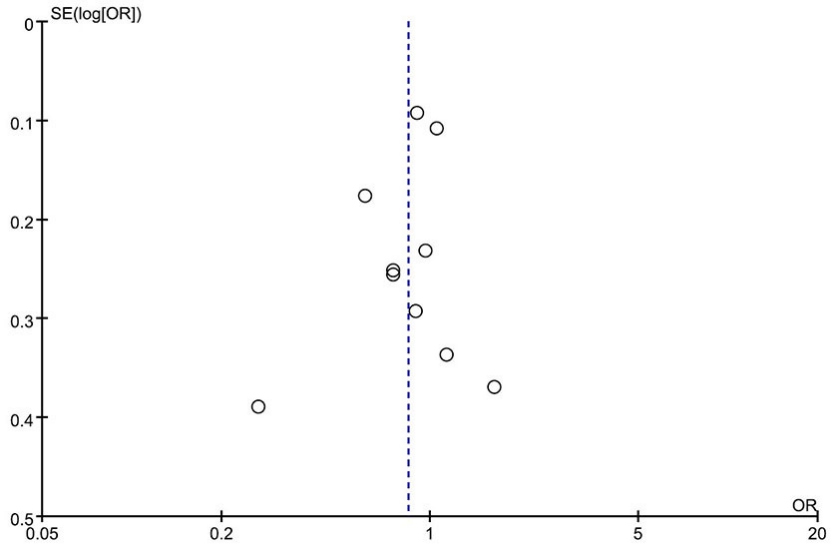
Funnel plot of G1246A polymorphism in the HCRTR2 gene and risk of CH.

Subgroup analysis by ethnicity (Asian and Caucasian) was performed to assess the correlation between rs2653349 and risk of CH in subjects of different ethnicities (Figure 4). The outcome showed that G1246A polymorphism and CH in Caucasian (OR: 0.89, 95% CI 0.77, 1.01; *p* = 0.08) and Asian populations (OR: 1.65, 95% CI 0.80, 3.41; *p* = 0.18) have no significant associations statistically. The leave-one-out sensitivity analysis was done, and the overall estimate remained unchanged after each included study was removed one by one (detailed data not shown). This confirmed that our conclusion was a robust and reliable one. The overall estimate remained unchanged after the removal of any included study (detailed data not shown). Interestingly, the heterogeneity across studies descended sharply after the removal of the study by Rainero et al. [2008] (*I*^2^ = 18%) (Figure 4). Therefore, the study by Rainero and colleagues was deemed as the major source of heterogeneity across studies. There was no obvious asymmetry of the funnel plot (Figure 5), and the Egger's test (*t* = 0.41, *p* = 0.696) and Begg's test (*z* = 0.36, *p* = 0.721) also suggested no significant publication bias.

### Meta-Regression

Meta-regression was carried out by residual (restricted) maximum likelihood (REML) with Knapp–Hartung modification to identify the possible source of variation among studies. Five different factors that may contribute to the heterogeneity between studies were tested: ethnicity (Asian or Caucasian), sample size (*n* < 500 or *n* ≥ 500), chronological order of publication (before or after 2010), methodological quality (moderate or high), and diagnostic criteria for CH (ICHD-II or ICHD-III). Intriguingly, sample size, methodological quality, and chronological order of publication all explained 100.00% of the variation observed across studies, but only chronological order of publication appeared to be significantly associated with the heterogeneity (*t* = 2.47, *p* = 0.039; residual *I*^2^ = 0%, adjusted *R*^2^ = 100%). Based upon the meta-regression analysis, ethnicity (*p* = 0.080) and diagnostic criteria for CH (*p* = 0.053) did not contribute significantly to the intrastudy variation (Table 3).

**Table 3:** Meta-regression analysis.

**Factor tested**	**Residual *I*^**2**^ (%)**	**Adjusted *R*^**2**^(%)**	***t***	***p***
Ethnicity (Asian or Caucasian)	6.76	13.38	−2.01	0.080
Sample size (*n* < 500 or *n* ≥ 500)	24.27	100.00	1.26	0.244
Chronological order of publication (before or after 2010)	0.00	100.00	2.47	0.039
Methodological quality (moderate or high)	25.29	100.00	−1.21	0.263
Diagnostic criteria for CH (ICHD-II or ICHD-III)	0.00	53.40	2.26	0.053

*CH, cluster headache; ICHD, International Classification of Headache Disorders*.

## Discussion

Although the etiopathogenesis of CH is complex and largely undetermined, both extrinsic and intrinsic factors are considered to contribute to the etiology. The *HCRTR2* gene being an intrinsic factor has gained increasing consideration and attention in the last few years. The correlation between G1246A polymorphism in the *HCRTR2* gene and CH vulnerability, which has been recently studied, gave inconsistence results. To prevent the shortcoming of deficient sample size and ethnic limitation in an individual study, we performed the current meta-analysis to evaluate the association of G1246A and CH with subjects of different ethnicities.

For the majority of patients, CH shows a distinct circadian and circannual regularity of the attacks, strongly suggesting involvement of the biological clock, which is regulated in the hypothalamic region of the brain (Barloese et al., 2015; Steinberg et al., 2018). Therefore, the hypothalamus has been a main focus of the pathophysiology of CH. The neuropeptide hypocretin 2 (HCRT2) is synthesized in the posterior hypothalamus and serves as ligands for G protein-coupled hypocretin receptors 2 (HCRTR2) (Sakurai et al., 1998). HCRT2 has been reported to affect nociceptive input and vasoregulation by modulation of sympathetic and parasympathetic responses (Dergacheva et al., 2005). The activation of the hypocretin receptor stimulation inhibits dural vasodilation upon electrical stimulation of trigeminal dural afferents (Holland et al., 2005). This might therefore provide a link to head pain and autonomic symptoms via the activation of *HCRTR2* (Bartsch et al., 2004). The mutation of V308I in the *HCRTR2* gene resulted in the substitution valine with isoleucine, thus the structure of *HCRT2* changed from an irregular curl to double helix structure, which further leads to the decrease of binding force between hypocretin and hypocretin receptors, and affects the downstream signal transduction (Rainero et al., 2008; Qianling, 2018). Rainero et al. [2008] reported that the mutation of the *HCRTR2* gene or a linked locus was significantly associated with the risk for CH. In addition, they also suggested that the V308I substitution of the *HCRTR2* may interfere with the dimerization process of the receptor, which thereby influences its functional activity. Schürks et al. [2006] observed that the G1246A polymorphism in the gene of the *HCRTR2* has been linked to the risk for CH. Compared to heterozygotes or homozygotes carrying the A allele, homozygotes carrying the G allele have a twofold increase in risk for CH (OR 1.97; 95% CI 1.32 to 2.92; *p* = 0.0007). These findings altogether demonstrated that *HCRTR2* genes participate and play an important role in CH pathogenesis.

However, our pooled data didn't support the association between G1246A polymorphism in the *HCRTR2* gene and CH (OR 0.85, 95% CI 0.69, 1.03; *p* = 0.10). This result was similar to previous studies. Baumber et al. [2006] suggested that there are no deleterious sequence variants of the *HCRTR2* gene by comparison to wild-type sequence, which is consistent with Zarrilli et al. [2015]. Weller et al. [2015] found no evidence for association between rs2653349 polymorphism in the *HCRTR2* gene and CH. This result is in agreement with studies performed in Sweden (Fourier et al., 2019), Southeastern European (Papasavva et al., 2020), and China (Fan et al., 2018). The possible reasons for negative results could be as follows: First, the results of previous published literature may be overestimated due to the limited sample size or different populations. Second, CH is a complex genetic disorder, which is inflected by multiple loci polymorphisms and a range of genes. Several candidate genes for CH have been investigated, such as ADH4 (Rainero et al., 2010), CACNA1A (Sjostrand et al., 2001), MTHFR (Schürks et al., 2011), and CLOCK (Fourier et al., 2018). However, the impact of a single loci polymorphism in the *HCRTR2* gene was considered and assessed in this study. This might hide the effect of G1246A polymorphism in the *HCRTR2* gene. Third, though the V308I substitution of the *HCRTR2* results in the substitution of valine with isoleucine, it didn't alter the activation of orexin-A-induced and orexin-B-induced extracellular signaling kinase (ERK1/2) in an *in vitro* study (Tang et al., 2008). Unfortunately, no published studies so far reported the association between G1246A polymorphism of the *HCRTR2* gene and its expression level or biological activity in humans. It is still unclear whether the G1246A necessarily contributes to the pathogenesis of CH.

Our result was inconsistent with the previous meta-analysis led by Rainero et al. [2007] and Weller et al. [2015]. There were several notable differences between our review and the two previous reviews: (1) Our review included several newly published studies with subjects beyond Caucasians and assessed the ethnicity-specific effect with association by subgroup analysis. (2) We performed quality assessment for each individual study according to the NOS. This ensured potential risk of bias was assessed and the level of evidence was evaluated in each study. (3) Our review included 5,125 subjects (1,964 CH patients and 3,161 healthy controls), which further powered our analysis. In contrast, the two previous meta-analyses had sample sizes of 1,336 and 2,785, respectively. (4) The stability and robustness of our outcome was evaluated and validated with sensitivity analysis. Consequently, the study led by Rainero et al. [2008] was regarded as having considerable heterogeneity. In comparison to other included studies, Rainero et al. included 109 CH patients and 211 healthy controls, which was smaller than other eligible studies. What's more, the frequency distribution of rs2653349 in the Southeastern European Caucasian population differs significantly with other European and East Asian populations (Katsarou et al., 2018). The study by Rainero et al. [2008] was performed in Italy, which is located in Southeastern European. This may cause the difference in ethnicity. In order to draw a more reliable conclusion, we excluded the study by Rainero et al. in our repeated meta-analysis, resulting in a significant decline of heterogeneity (*I*^2^ = 18%) (Figure 4). (5) The results of a meta-regression showed that the chronological order of publication significantly contributed to the intrastudy heterogeneity (residual *I*^2^ = 0%, *p* = 0.039), suggesting that chronological order of publication could be a possible source of intrastudy variability. It could be possible that the conclusions of first-published studies have important implications for the pursuit of research on the given association. Statistically significant first-published articles are more attractive and are more likely to be followed by more studies over time, whereas initially non-significant results do not attract further investigation. It should be noted that although the relatively small sample size of included studies (*n* < 500) was not statistically associated with the intrastudy variability, it explained part of the heterogeneity among studies (residual *I*^2^ = 24.27, *p* = 0.244), which again highlighted the need for studies with larger sample sizes to detect the modest genetic effect of *HCRTR2* polymorphisms.

There were several limitations in this review that should not be neglected. First, the sample size of all included studies was hundreds of cases and controls. Studies with thousands of cases and controls could provide better evidence to identify the marginal effect of correlation between the *HCRTR2* gene and risk of CH. Second, seven out of the eight studies were hospital-based. This might exaggerate OR values caused by a high selection of subjects. Third, two causes were considered to account for the null association of our present study. First, according to our results of meta-regression, the sample size (*n* < 500) appeared to be the source of heterogeneity among included studies. The limited sample size of our included studies was not vigorous enough to obtain a significant association. Second, the frequency distribution of rs2653349 showed a statistically significant difference between Southeastern European Caucasian and other European and East Asian populations (Katsarou et al., 2018). Participants among those included studies have different ethnicities. This demographic heterogeneity might also conceal the real association. Nonetheless, even if our present study has some shortcomings, the methodological quality overall of the included studies was moderate. A reasonable degree of confidence should be granted to the null association between G1246A polymorphism in the *HCRTR2* gene and risk of CH based upon the results of our meta-analysis. Further association studies with larger sample size are strongly encouraged.

## Conclusion

Collectively, the combined results didn't support the association between G1246A polymorphism in the *HCRTR2* gene and CH vulnerability across both Caucasian and Asian ethnics. Concerning limitations of the current study, our findings need further confirmation by well-designed and population-based investigations with larger sample sizes among more ethnicities.

## Data Availability Statement

The original contributions presented in the study are included in the article/Supplementary Materials, further inquiries can be directed to the corresponding author/s.

## Author Contributions

S-yY and Z-tL designed and supervised the research. JiaY, JK, and JieY participated in the acquisition of data and performed the meta-analyses. JiaY and Z-tL helped draft the manuscript. F-rL and Z-tL revised the manuscript. All authors reviewed and approved the manuscript.

## Conflict of Interest

The authors declare that the research was conducted in the absence of any commercial or financial relationships that could be construed as a potential conflict of interest.

## Supplementary Material

Supplementary File 1 Funding support document.

Supplementary File 2 MOOSE checklist.

## Abbreviations

HCRTR2: hypocretin receptor type 2
CH: cluster headache
NOS: Newcastle–Ottawa Scale
ICHD-III: International Classification of Headache Disorders, 3rd edition
GNB3: G protein beta3 subunit
ADH4: alcohol dehydrogenase 4
PPI: protein–protein interaction
NPFF: neuropeptide FF-amide peptide
HCRT: hypocretin
ICHD-II: International Classification of Headache Disorders, 2nd edition
ICHD-III beta: International Classification of Headache Disorders
HWE: Hardy–Weinberg equilibrium.
