# Supplementary material for: No Association Between G1246A Polymorphism in HCRTR2 Gene and Risk of Cluster Headache: Evidence From an Updated Meta-Analysis of Observational Studies
Source: Front Genet. 2020 Dec 3;11:560517. doi: 10.3389/fgene.2020.560517 (PMC7744679; doi:10.3389/fgene.2020.560517)
Supplement: Supplementary File 1 — Funding support document. [file Data_Sheet_1.PDF]

项目批准号	81590951
申请代码	H2707
归口管理部门	
依托单位代码	61113708A0136-0226

815909511015508

国家自然科学基金委员会

资助项目计划书

资助类别：重大项目

亚类说明：课题申请

附注说明：穴位的敏化研究

项目名称：穴位敏化现象和规律的临床研究

直接费用：330万元　　间接费用：64.284万元

项目资金：394.284万元　　执行年限：2016.01-2020.12

负 责 人：梁繁荣

通讯地址：成都市温江区柳台大道1166号

邮政编码：611137　　电　　话：028-61800006

电子邮件：lfr@cdutcm.edu.cn

依托单位：成都中医药大学

联 系 人：李炜弘　　电　　话：028-61800101

填表日期：2015年12月01日

Approved number	81590951
Application code	H2707
Designated manage ment organization	
Dependent unit code	61113708A0136-0226

815909511015508

National Nature Science Foundation of China

Project proposal

Funding category: National Nature Science Foundation of major project in China

Subclass interpretation: Project application

Notes Explanation: Research of acupoint sensitization

Project Name: Clinical study of the regularity and phenomenon of acupoint sensitization

Direct Costs : ￥3.3million **Indirect Costs:** ￥0.64284million

Project Costs: ￥3.94284million **Execute time:** 2016.01-2020.12

Person in Charge: Fanrong Liang

Correspondence Address: 1166 Liutai avenue, wenjiang district, Chengdu city

Post code: 611137 **Telephone Number:** 028-61800006

Email Address: lfr@cdutcm.edu.cn

Affiliation: Chengdu University of Traditional Chinese Medicine

Contact Person: Hongwei Li **Telephone Number:** 028-61800101

Date of Filling: December 1, 2015

National natural science foundation of China

Version: 1.015.508
